# Accuracy and Safety of Robot-Assisted versus Fluoroscopy-Guided Posterior C1 Lateral Mass and C2 Pedicle Screw Internal Fixation for Atlantoaxial Dislocation: A Preliminary Study

**DOI:** 10.1155/2022/8508113

**Published:** 2022-09-12

**Authors:** Jiheng Zhan, Wenke Xu, Jinhao Lin, Jiyao Luan, Yu Hou, Yufeng Wang, Yongjin Li, Bolai Chen, Dingkun Lin, Shudong Chen

**Affiliations:** ^1^Department of orthopedics, Guangdong Provincial Hospital of Chinese Medicine, Guangzhou 510120, China; ^2^Postdoctoral Workstation, Guangdong Provincial Hospital of Traditional Chinese Medicine, Guangzhou 510120, China; ^3^Research Team on the Prevention and Treatment of Spinal Degenerative Disease, Guangdong Provincial Academy of Chinese Medical Sciences, Guangzhou 510006, China; ^4^Postdoctoral Research Station, Chinese Academy of Chinese Medical Sciences, Beijing 100700, China; ^5^Second Clinical College, Guangzhou University of Chinese Medicine, Guangzhou 510405, China

## Abstract

**Objective:**

To compare the accuracy, efficiency, and safety of robotic assistance (RA) and conventional fluoroscopy guidance for the placement of C1 lateral mass and C2 pedicle screws in posterior atlantoaxial fusion.

**Methods:**

The data of patients who underwent posterior C1–C2 screw fixation (Goel-Harm's technique) in our hospital from August 2014 to March 2021 were retrospectively evaluated, including 14 cases under fluoroscopic guidance and 11 cases under RA. The hospital records, radiographic results, surgical data, and follow-up records were reviewed. Accuracy of screw placement was assessed using the Gertzbein and Robbins scale, and clinical outcomes were evaluated by Japanese Orthopedic Association (JOA) score, visual analogue scale (VAS), modified MacNab criteria, and postoperative complications.

**Results:**

Baseline characteristics of both groups were similar. The mean estimated blood loss in the fluoroscopic guidance and RA groups was 205.7 ± 80.3 mL and 120.9 ± 31.9 mL, respectively (*p* = 0.03). The mean surgical duration was 34 min longer with RA compared to that performed with free-hand (FH) method (*p* = 0.15). In addition, lower intraoperative radiation exposure was detected in the RA group (12.4 ± 1.4 mGy/screw) versus the FH (19.9 ± 2.1 mGy/screw) group (*p* = 0.01). The proportion of “clinically acceptable” screws (graded 0 and I) was higher in the RA group (93.2%) than that in the FH group (87.5%, *p* = 0.04). There was no significant difference in the increase of JOA score and decrease of VAS score between the two surgical procedures. Furthermore, there were no significant differences in overall clinical outcome between the two groups and no neurovascular complications associated with screw insertion.

**Conclusions:**

RA is a safe and potentially more accurate alternative to the conventional fluoroscopic-guided FH technique for posterior atlantoaxial internal fixation.

## 1. Introduction

The atlantoaxial junction is a specialized region that plays an important role in the movements at the cervico-vertebral joint. Although the atlantoaxial junction is one of the most stable joints in the body, it remains vulnerable to trauma, rheumatoid arthritis, infections, congenital malformations, and malignancies [1]. The resulting deterioration of the zygapophyseal joint, ligament, or muscles between the atlas (C1) and axis (C2) can cause excessive movement and instability at this junction, resulting in atlantoaxial dislocation (AAD). It can cause neck pain and spinal cord compression, and even irreversible neurological deficits, such as cervical myelopathy, paresis, and respiratory dysfunction. For AAD patients with persistent neck pain and neurologic symptoms, surgical intervention is a good choice. Many different surgical techniques have been developed to stabilize the atlantoaxial complex and achieve spinal cord decompression. Currently, Goel-Harm's technique of C1 lateral mass screw (C1LMS) and C2 pedicle screw (C2PS) is the preferred one because of the lower risk of vertebral artery (VA) injury and minimal damage to facet joints [2]. However, the anatomy of the upper cervical spine is highly variable, and the presence of peripheral neurovascular abnormalities makes atlantoaxial arthrodesis technically challenging [3]. The mean incidence of VA injury caused by misplacement of pedicle screws during atlantoaxial fusion was 8.2% to 21.6% under intraoperative biplane fluoroscopy [4, 5]. To avoid these complications, several advanced techniques have been introduced for assisting screw placement during upper cervical fixation [6–8].

Surgical robotics is widely applied in general surgery, urology, neurosurgery, and orthopedics [9]. In thoracolumbar surgery, robotic assistance (RA) has been shown to improve screw placement accuracy, reduce surgical bleeding, and minimize intraoperative radiation [7, 10]. Nevertheless, there are no reports evaluating the accuracy, efficiency, and safety of robot-assisted posterior atlantoaxial arthrodesis due to the lack of specialized robotic systems for cervical spine surgery. Our medical center installed the intraoperative 3D image-based TiRobot system for orthopedic surgery in 2018. In this study, we have briefly described the robotic structures and workflow and presented the preliminary results of a retrospective study comparing the radiological and clinical results of C1–C2 screw placement using fluoroscopy or RA.

## 2. Materials and Methods

### 2.1. Patient Demographics

The study protocol was approved by the ethics committees of Guangdong Provincial Hospital of Chinese Medicine, and the requirement for informed consent was waived due to the retrospective nature of this study. Consecutive patients were diagnosed with AAD and surgically treated with posterior C1–C2 screw fixation under fluoroscopic guidance or RA at our hospital from August 2014 to March 2021. All procedures were performed by the same surgical team. The clinical variables including age, gender, body mass index (BMI), and medical history (symptom duration and pathology) are summarized in Table 1.

### 2.2. Surgical Techniques

Based on the guiding technique for screw placement, the patients were divided into the conventional fluoroscopy-guided free-hand (FH) group and the RA group using intraoperative robotic navigation devices. Our institute installed TiRobot (co-designed by Beijing Jishuitan Hospital and TINAVI Medical Technologies Co., Ltd.), a novel system consisting of a robotic arm, an optical tracking system, a robotic workstation, and a navigation toolkit for posterior atlantoaxial internal fixation based on intraoperative 3D images. In order to detect neurological function and avoid spinal injury, motor evoked potentials (MEPs) and somatosensory evoked potentials (SEPs) were monitored throughout surgery.

#### 2.2.1. Fluoroscopy-Guided FH Surgery

Following general anesthesia, the patient was placed in the prone position with a radiolucent Mayfield clamp to keep the neck slightly flexed. Then, a posterior median incision was made from the base of the skull to C4. After exposing the posterior arch of C1, the lateral of C1, and the C1–C2 facet joint, fixation was performed with Goel-Harm's technique [11]. Using direct visualization and anteroposterior and lateral fluoroscopy (BV Endura; Philips, Eindhoven, Netherlands), starting holes were made in the lateral mass of C1 and in the pedicle of C2, respectively. Subsequently, the pilot hole is drilled under fluoroscopic guidance and verification of depth with a blunt probe. After that, the screws (DePuy Synthes, Raynham, Massachusetts, USA) were implanted under X-ray guidance, followed by the placement of 2 rods of appropriate length for connecting C1 and C2. Finally, the spinal canal was decompressed, and posterolateral fusion was performed with iliac crest bone grafting.

#### 2.2.2. RA Surgery

**Figure 1:**
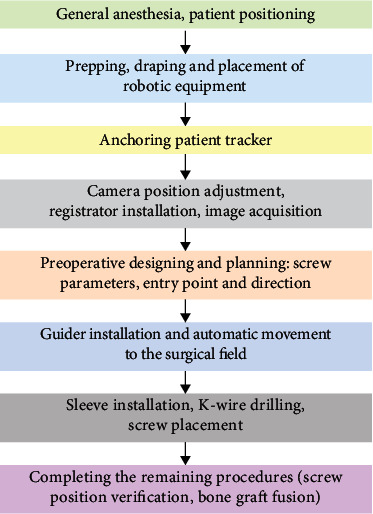
Workflow of robot-assisted procedures.

Robot-assisted posterior atlantoaxial internal fixation was performed as previously described by Tian et al. [12] with minor modifications (Figure 1). The TiRobot system was arranged in the operating room as shown in Figure S1. The patients were anesthetized and positioned as in traditional surgery. The patient tracker was securely anchored onto the Mayfield head frame, and the registration was installed and placed into the operating area. The posterior region of the C1–C2 complex was exposed via a midline incision in a standard subperiosteal manner. Following adjustment of the camera position towards the operation space and the patient tracker, the registration was installed and placed into the operating area. A set of images were obtained by intraoperative 3D C-arm scanning, and the trajectories, diameters, and lengths of C1LMS and C2PS were planned according to these images in the robotic station. Subsequently, the robotic arm was moved automatically to guide the planned trajectory, and a cannula was inserted through the sleeve of the robotic arm, followed by drilling of the K-wire into the vertebrae. Following the verification of the optimal path, a cannulated screw was instrumented along the K-wire and the other three screws were inserted in the same manner. The screw position was confirmed, followed by screw-rod connection and bone graft fusion.

### 2.3. Assessment of Surgical Data

Surgery-related parameters including surgical duration, EBL, radiation dosage, and fusion rate were recorded, along with the length of postoperative hospital stay and follow-up. Vascular and neural complications associated with screw insertion were also evaluated.

### 2.4. Evaluation of Screw Placement Accuracy

Computed tomography (CT) scan with multiplanar reconstructions of the cervical spine was performed 2 days after surgery to determine the accuracy of screw placement. Two independent observers evaluated the CT images in a blinded manner to determine the number of misplaced screws and the grade of screw placement. The accuracy of screw placement was assessed using a grading system proposed by Gertzbein and Robbins [13]: grade 0, when a screw was completely placed inside the bone; grade I, screw perforation of the cortex <2 mm; grade II, screw perforation ≥2 but <4 mm; and grade III, screw perforation ≥4 mm (Figure S2). Screws graded 0 and I are clinically acceptable, while those graded II and III are significantly deviated from the intended trajectory and can potentially cause neurovascular damage. CT angiography (CTA) or magnetic resonance angiography (MRA) was immediately performed for patients with malpositioned screws to assess potential VA injury.

### 2.5. Clinical Recovery Assessment

Functional outcomes were assessed 1 week, 1 month, 3 months, and 6 months postsurgery in terms of Japanese Orthopaedic Association (JOA) and visual analogue scale (VAS) scores. The satisfaction rate of clinical outcomes at the final follow-up was assessed according to the modified MacNab criteria (excellent, good, fair, or poor). The scores were calculated by two trained observers. In case of disagreement, a third senior surgeon was asked to assist in the evaluation.

### 2.6. Statistical Analysis

Statistical analyses were performed using the statistical package SPSS version 20.0 (IBM Corporation, Somers, NY, USA). All continuous variables were presented as mean ± standard deviation, and the proportions were expressed as numbers (%). The Shapiro-Wilk test was applied to check the normality of data. Paired *t*-test was used for the preoperative and follow-up parameters (JOA and VAS). Independent *t*-tests were used for the surgical results and postoperative imaging measurements of the 2 different procedures. The descriptive assessments and analytical statistics of enumeration data were performed depending on the group characteristics. Statistical significance (*p* value) was set at 0.05.

## 3. Results

### 3.1. Demographic and Clinical Characteristics

From August 2014 to March 2021, a total of 25 patients underwent posterior C1–C2 screw fixation, including 14 with fluoroscopy-guided FH surgery (55.4 ± 14.6 years) and 11 RA surgery (50.6 ± 17.7 years). The follow-up rate was 96.2% since 1 patient in the FH group was lost during that period. As shown in Table 1, most baseline characteristics did not differ between the FH and RA groups, and the gender distribution was uniform in both groups.

### 3.2. Comparison of Surgical Results between the Two Groups

**Figure 2:**
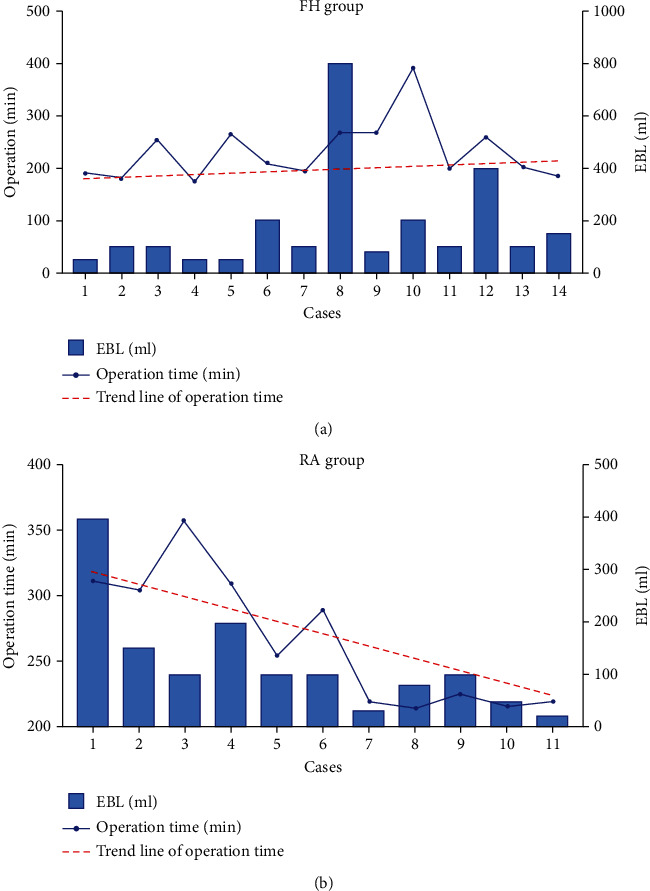
Impact of surgeon's experience on surgery parameters. (a) In the FH group, the EBL varied widely from case to case and the trend line of the surgical duration did not change significantly with time. (b) In the RA group, the trend line of the surgical duration decreased and EBL was stabilized with more operations.

The intraoperative period was uneventful in all patients. Mean EBL was 205.7 ± 80.3 mL in the FH group, almost double that of the RA group (120.9 ± 31.9 mL, *p* = 0.03; Table 2). It is worth noting that RA was associated with a slight increase in the length of procedure (*p* = 0.15; Table 2). On average, operations with RA lasted 266.0 ± 15.3 min, while operations under conventional fluoroscopy guidance lasted 232.3 ± 15.8 min. Reassuringly, the duration of surgery in the RA group showed a decreasing trend as more surgeries were performed, suggesting a learning curve effect, which was confirmed by linear regression (*R*^2^ = 0.6367; Figure 2). There was no significant difference in the length of postoperative hospital stay between the two groups. A major concern of image-guided surgery is radiation exposure to patients and operating room personnel. The average intraoperative dosage of radiation exposure in the FH group was 19.9 ± 2.1 mGy/screw, while that of the RA group was significantly reduced to 12.4 ± 1.4 mGy/screw (*t* = 2.78, *p* = 0.01).

### 3.3. Comparison of the Screw Placement Accuracy

A total of 100 screws, including 50 C1LMS and 50 C2PS, were placed and analyzed using thin-slice CT scan. The accuracy of screw positions is summarized in Table 3. In the RA group, a completely intrabone course (grade 0) was found in 84.1% (*n* = 37) of the screws. The remaining screw placements were graded I (*n* = 4, 9.1%) and II (*n* = 3, 6.8%). In the FH group, a perfect trajectory (grade 0) was observed in 41 screws (73.2%), and the remaining were graded I (*n* = 8, 13.3%), II (*n* = 5, 8.9%), and III (*n* = 2, 3.6%). Furthermore, the “clinically acceptable” rate of screw placement in the RA group was 93.2% compared to 87.5% in the FH group (*p* = 0.04).

### 3.4. Comparison of the Clinical Recovery Outcomes

**Figure 3:**
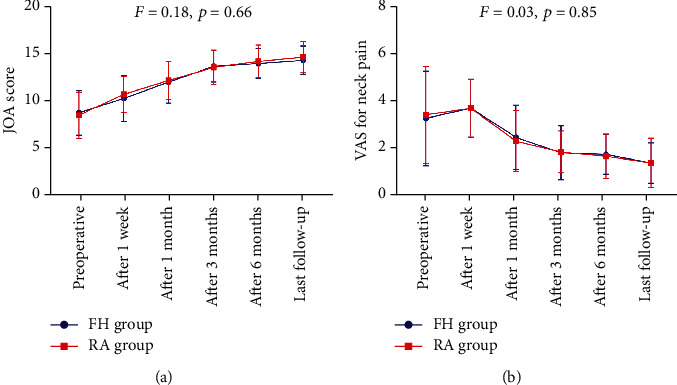
Comparisons of the improvement of JOA and VAS scores between the FH and RA groups. There were no significant differences in the postoperative JOA scores and VAS scores of neck pain relative to preoperative values between the two procedures.

Patients were followed for a median period of 11.56 months (range 6–24 months). As shown in Figure 3, both groups showed significant improvements in JOA scores at all follow-up time points in comparison to baseline levels. The improvement rate of JOA score was 64.06 ± 11.34% and 70.78 ± 21.36% in the FH and RA groups, respectively, with no statistical significance (*t* = 1.01, *p* = 0.32). The VAS scores in both groups increased slightly 1 week after surgery compared to the preoperative values but decreased gradually at the subsequent follow-up phases. However, there were no significant differences in the JOA scores and VAS scores of neck pain between the two groups at any follow-up time point (*p* = 0.66 and *p* = 0.85, respectively), indicating that both surgical procedures can improve postoperative functional recovery and relieve pain. The overall excellent and good rate was 92% (excellent in 9 patients, good in 3 patients, fair in 2 patients, and no poor patient). There were no significant differences in the satisfaction rates (excellent and good) between the FH and RA groups (85.7% versus 100%, *p* = 0.39).

Serious complications such as neurovascular injury or cerebrospinal fluid leakage due to the screw fixations were not observed in either group. No wound hematomas, infections, and other perioperative complications were found. Only one case of postoperative delirium was observed in the FH group, and the symptoms were recovered spontaneously after 24 hours.

## 4. Discussion

Vital structures such as the vertebral arteries and spinal cord are in close proximity to the atlantoaxial joint. The axis vertebra demonstrates high anatomical variations such as narrow pedicle sizes and high-riding VA (HRVA) [14]. As a result, screw placement is a highly risky procedure that may result in screw perforations and increase the risk of neurovascular injury [15, 16]. For spinal surgery, accurate screw placement depends on the selection of correct insertion point, monitoring of the trajectory position, and the surgeons' skills. However, surgeries involving the cervical spine are prolonged and tedious, which can lead to both mental and physical fatigue, and increase the risk of potentially catastrophic errors. Furthermore, traditional posterior atlantoaxial screw placement requires extensive soft-tissue dissection to distinguish the anatomical features and repeated use of the C-arm X-ray machine to confirm the position of screws. Therefore, it is crucial to develop a procedure that is minimally invasive and can achieve high screw placement accuracy with lower intraoperative radiation exposure. Costa et al. [17] reported that 92.6% of the screws were correctly placed in 17 patients with C1–C2 traumatic fractures who underwent surgery guided by the intraoperative 3D image-based navigation system. Jacobs et al. [18] also presented similar results and suggested that intraoperative CT-based navigation is a viable tool for detecting screw misplacement. Although navigation improves the accuracy, it might require repeated adjustments of the trajectories [19] when inserting screws, which is inconvenient and increases the risk of C2 nerve stimulation and spinal canal invasion.

Robotic systems can perform repetitive tasks with precision and reproducibility, making them ideal for spinal surgery. In the last few decades, nearly 20 robotic systems have been developed for spinal surgery and tested in clinical studies, including Spine Assist (Mazor Robotics Inc®, Orlando, Florida), ROSA (Medtech S.A., Montpellier, France), and Da Vinci Surgical System (Intuitive Surgical). However, these systems can only be used for thoracic and lumbar surgery due to their bone-mounted design and inability to meet the screw placement accuracy required for cervical surgery [9]. TiRobot is currently the only orthopedic robot system that can be used for posterior screw insertion in the cranio-cervical junction area [7, 8, 20]. Although there are case reports of the TiRobot system for C1–C2 transarticular screw fixation and anterior odontoid screw fixation [8, 21], studies evaluating radiological results and clinical outcomes of robotic-assisted posterior C1–C2 fixation (Goel-Harm's technique) are lacking. In this study, we compared the accuracy, efficiency, and safety of screw placement with the TiRobot system and biplane X-ray fluoroscopy for posterior atlantoaxial internal fixation. Consistent with a previous study [22], the proportion of “clinically acceptable” screws (graded 0 and I) was 93.2% with RA compared to 87.5% in the FH group. Out of the 100 inserted screws, ten were malpositioned (graded II and III) but did not result in severe neurovascular injury, cerebrospinal fluid leakage, or new neurologic defects.

Higher image resolution and more accurate localization with advanced imaging equipment are often accompanied by greater radiation exposure. Radiation exposure to the surgeon, patient, and operating room staff can be significant, especially in multi-segmental fusion and revision surgery, in which patients have distorted anatomy and no longer possess regular anatomic landmarks [23]. The radiation doses are 10- to 12-fold higher during spinal surgeries compared to that measured during nonspinal procedures [24]. Additionally, minimally invasive surgery (MIS) techniques cause even more radiation exposure because bony landmarks are obscured and can only be detected by fluoroscopy [25, 26]. Orthopedic surgeons who are routinely exposed to radiation have a significantly increased risk of cancer [27]. However, intraoperative electromagnetic navigation and robotic guidance system can potentially lower the dose of radiation exposure [28–30]. Schoenmayr and Kim [31] reported a 60% reduction in median radiation exposure with robot-assisted percutaneous spinal fusion surgery compared to conventional pedicle screw insertion techniques. Similarly, another single-center study showed that radiation exposure under Spine Assist guidance was 70% lower than that of conventional procedures [32]. The radiation exposure for RA surgeries in our study was also less compared to that in FH procedures. Furthermore, the average intraoperative radiation dosage of the first three cases was relatively high (17.7 ± 2.9 mGy/screw), but the overall radiation dosage gradually decreased as the number of cases increased.

Significant blood loss and need for transfusion carry many risks including infection and coagulopathy, especially in trauma patients and in elderly patients with other comorbidities [33]. In this series, we observed a significant reduction in the amount of intraoperative blood loss when the TiRobot system was used during atlantoaxial fixation. This finding is likely explained by the optimization of surgical exposure of the C1–C2 articular complex, thereby minimizing disturbance of the robust cervical venous plexuses. In addition, the TiRobot system enables more precise intraoperative image guidance during hardware placement, thereby improving the chance of successful screw placement at one time and avoiding excessive bleeding due to the repeated adjustment of screw channels, especially in the case of C2 segment HRVA. Although EBL was relatively high and varied widely in the first few patients in the RA group (range from 100.0 to 400.0 mL), EBL decreased from 190.0 ± 111.4 mL in the first 5 cases to 56.0 ± 30.1 mL in the last 5 cases, accompanied by a decrease in surgical duration.

**Figure 4:**
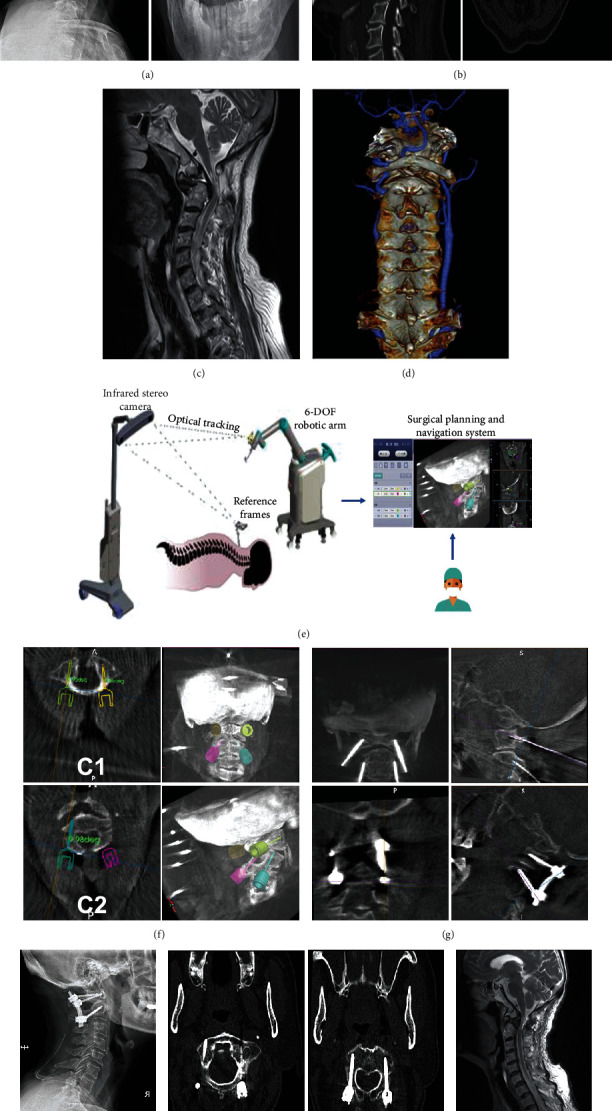
Case presentation. A 65-year-old female patient presented with a 3-year-long history of progressive dizzy, numbness, and weakness of extremities. (a, b) X-ray and CT examinations indicated os odontoideum and atlantoaxial instability (white arrows). (c) Preoperative magnetic resonance imaging (MRI) indicated a high signal intensity in the medulla oblongata. (d) No changes in VA course were observed in the 3D-CTA images. (e) The working principles of the TiRobot system. (f) Surgical planning of trajectories in the TiRobot system. (g) Robot-assisted insertion of C1LMS and C2PS. (h–j) X-ray, CT, and MRI images at 3 months postsurgery demonstrated that dislocation was repaired, compression was released, and there were no perforations of the screws.

Unlike other robotic systems, TiRobot is built with an image-navigated robotic positioning platform including a robotic arm, an optical tracking system, and a surgical planning and navigation system (Figure 4(e)). The integration of robotic arm and navigation system allows seamless flow of information between planning and execution of the surgery [7]. Furthermore, the optical tracking system can detect the actual patient position and subtle postural changes in real time and coordinate with the robotic arm for real-time motion compensation, so that the arm is always accurately positioned in the preplanned screw placement trajectory. Additionally, the TiRobot system can also be used for the surgeries of long bones, femoral neck and pelvis, etc., with different hardware modules [34–37].

Although no serious complications were observed in our cohort, the risk of screw misplacement is substantial due to multiple causes, such as excessive pulling of the soft tissue during exposure and spinal destabilization due to extensive decompression or osteotomy. This results in a large relative displacement between the bony structures and the patient tracker. Accidental displacement of the tracker during surgery can decrease positioning accuracy or even lead to failed positioning. The robotic system must maintain good reflection and reception of infrared light. Misalignment may occur if the angle or distance exceeds the receiving range or in case of any other light interference [12]. In the case of displacement of the patient tracker, there can be gross neurovascular injury if the incorrect trajectory is followed. We also noted skidding of the drill cannula, which was attributed to skidding of the sleeve tip on the hard cortical bone surface lateral to the facet joints. Therefore, it is important for a surgeon not to rely entirely on the robot but also to use his skill and experience. When necessary, intraoperative fluoroscopy should be performed to avoid catastrophic complications. In our study, RA shortened the duration of surgery as the number of surgical cases increased, again highlighting the importance of the surgeon's experience. Increased experience and familiarity of the operative team with the robotic system and logistics could result in a decrement of procedure length over time. A learning curve of 30 cases using RA is estimated to avoid screw malposition [30]. Similarly, Schatlo, Martinez, and Alaid suggested a minimum of 25 procedures for increasing the accuracy of robotics in spinal surgery [38]. However, the more recent generation seems to be less demanding in terms of technique [39].

## 5. Limitations

Our study had several inherent limitations. First, biases are common in single-center retrospective comparative studies, which affect the conclusion. Second, the sample size was comparatively small which might limit the generalizability of the findings. Third, with limited follow-up time, we can only compare the early results of the two procedures. These factors may influence our results and interpretation. Therefore, a longer follow-up, randomized case-control and multicenter study will be needed in the future.

## 6. Conclusion

This study demonstrated that compared to the conventional fluoroscopy-guided FH surgery, robotically assisted surgery resulted in minimal trauma, reduced bleeding and less intraoperative radiation exposure, and improved screw placement accuracy in AAD patients. The TiRobot system is still in its early developmental stage and may revolutionize spinal surgeries following improvements.

## Figures and Tables

**Table 1:** Comparison of the patients' demographic characteristics.

	FH group (*n* = 14)	RA group (*n* = 11)	*p* − value
Age (years)	55.4 ± 14.6	50.6 ± 17.7	0.46
Gender (*n*)			
Male	10	6	0.38
Female	4	5	
BMI (kg/m^2^)	21.3 ± 2.8	23.3 ± 2.5	0.302
Symptom duration (months)	17.6 ± 4.9	27.3 ± 11.5	0.41
Postoperative hospital stay (days)	11.2 ± 5.0	9.4 ± 4.8	0.38
Follow-up time (months)	12.2 ± 1.3	10.73 ± 1.4	0.45
Pre-JOA	8.71 ± 2.33	8.45 ± 2.42	0.79
Pre-ODI	3.21 ± 1.97	3.36 ± 2.01	0.85
Pathology			
Trauma	5	4	0.98
Degeneration	3	2	
Congenital malformation	3	3	
RA	3	2	
Tumor	0	0	

Data are presented as mean ± SD or number (%) unless otherwise indicated. SD: standard deviation; BMI: body mass index; RA: rheumatoid arthritis.

**Table 2:** Comparison of patients' operative parameters.

Parameters	FH group (*n* = 14)	RA group (*n* = 11)	*p* − value
EBL (ml)	205.7 ± 80.3	120.9 ± 31.9	0.03^∗^
Surgical duration (min)	232.3 ± 15.8	266.0 ± 15.3	0.15
Radiation dosage (mGy/screw)	19.9 ± 2.1	12.4 ± 1.4	0.01^∗^
Fusion rate, unfused/fused (%)	1/13 (92.9)	0/11 (100)	0.37

Data are presented as mean ± SD or number (%) unless otherwise indicated. EBL: estimated blood loss. ^∗^*p* value < 0.05 was considered to be statistically significant.

**Table 3:** Accuracy of the screw position according to the grading system.

	Grade 0	Grade I	Grade II	Grade III
FH group				
C1LMS (28 screws)	21	4	2	1
C2PS (28 screws)	20	4	3	1
Total (56 screws)	41	8	5	2
Accuracy rate (%)	73.2	14.3	8.9	3.6
RA group				
C1LMS (22 screws)	18	3	1	0
C2PS (22 screws)	19	1	2	0
Total (44 screws)	37	4	3	0
Accuracy rate (%)	84.1	9.1	6.8	0

## Data Availability

The datasets used and/or analyzed to support the findings of this study are available from the corresponding author upon reasonable request.

## Abbreviations

AAD: Atlantoaxial dislocation
RA: Robotic assistance
FH: Free-hand
VA: Vertebral artery
C1LMS: C1 lateral mass screw
C2PS: C2 pedicle screw
CT: Computed tomography
CTA: CT angiographic
MRI: Magnetic resonance imaging
MRA: Magnetic resonance angiography
EBL: Estimated blood loss
JOA: Japanese Orthopaedic Association
VAS: Visual analogue scale
BMI: Body mass index.

## Supplementary Materials

Supplementary Materials Figure S1: schematic diagram of operation room. Supplemental Figure S2: the position of the screw placement using a grading system proposed by Gertzbein and Robbins. Grade 0, when a screw was completely placed inside the bone; grade I, screw perforation of the cortex <2 mm; grade II, screw perforation ≥2 but <4 mm; and grade III, screw perforation ≥4 mm.
