# Supplementary material for: Accuracy and Safety of Robot-Assisted versus Fluoroscopy-Guided Posterior C1 Lateral Mass and C2 Pedicle Screw Internal Fixation for Atlantoaxial Dislocation: A Preliminary Study
Source: Biomed Res Int. 2022 Sep 12;2022:8508113. doi: 10.1155/2022/8508113 (PMC9484877; doi:10.1155/2022/8508113)
Supplement: Supplementary Materials — Figure S1: schematic diagram of operation room. Supplemental Figure S2: the position of the screw placement using a grading system proposed by Gertzbein and Robbins. Grade 0, when a screw was completely placed inside the bone; grade I, screw perforation of the cortex <2 mm; grade II, screw perforation ≥2 but <4 mm; and grade III, screw perforation ≥4 mm. [file 8508113.f1.docx]

**Supplemental figures and figure legends**

**Figure S1.**

**
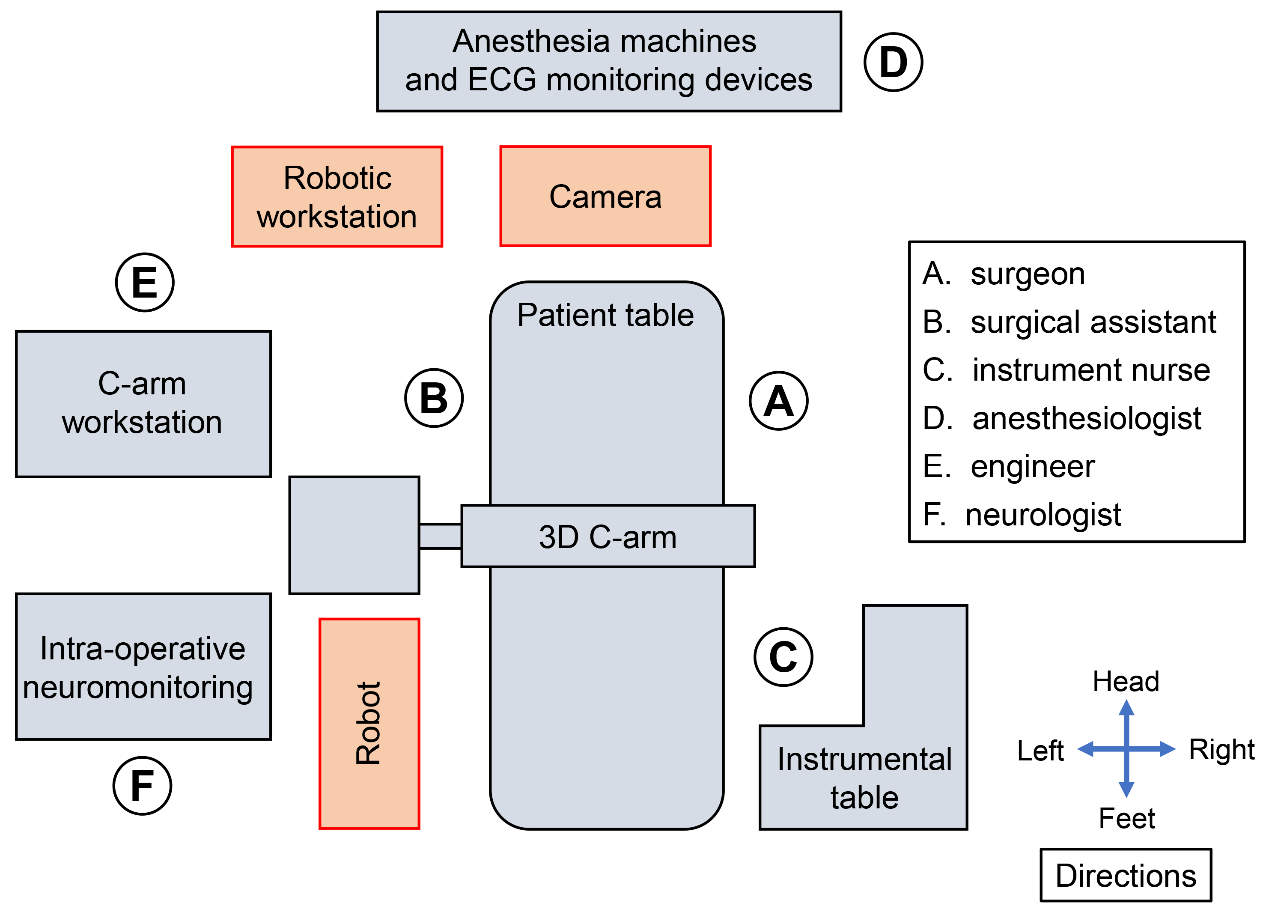
**

**Figure. S1** Schematic diagram of operation room. When performing robotic surgery, it is recommended that the operation room be arranged as shown.

**Figure S2.**


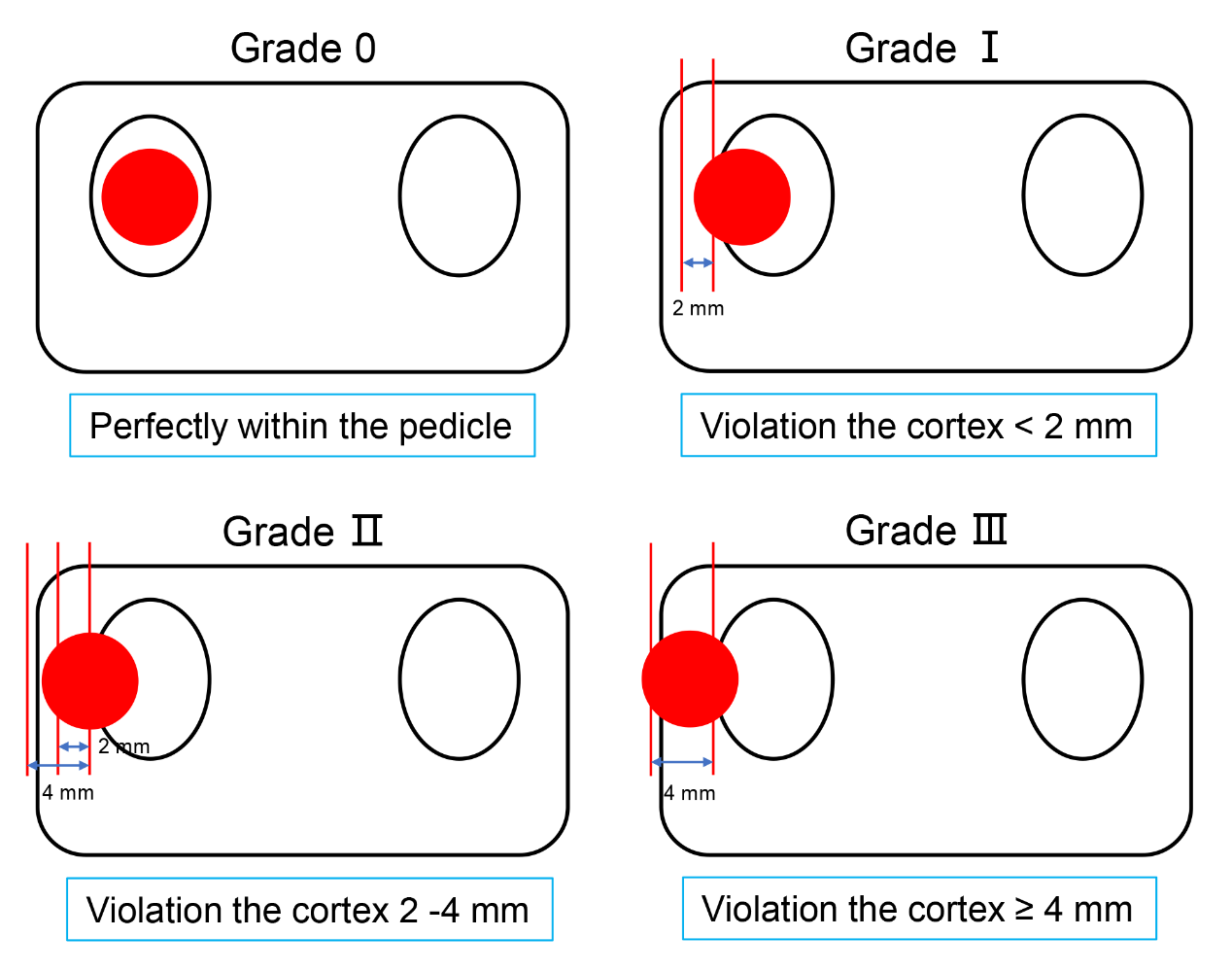
 **Figure. S2** The position of the screw placement using a grading system proposed by Gertzbein and Robbins. Grade 0, when a screw was completely placed inside the bone; grade I, screw perforation of the cortex < 2 mm; grade II, screw perforation ≥ 2 but < 4 mm; and grade III, screw perforation ≥ 4 mm.
